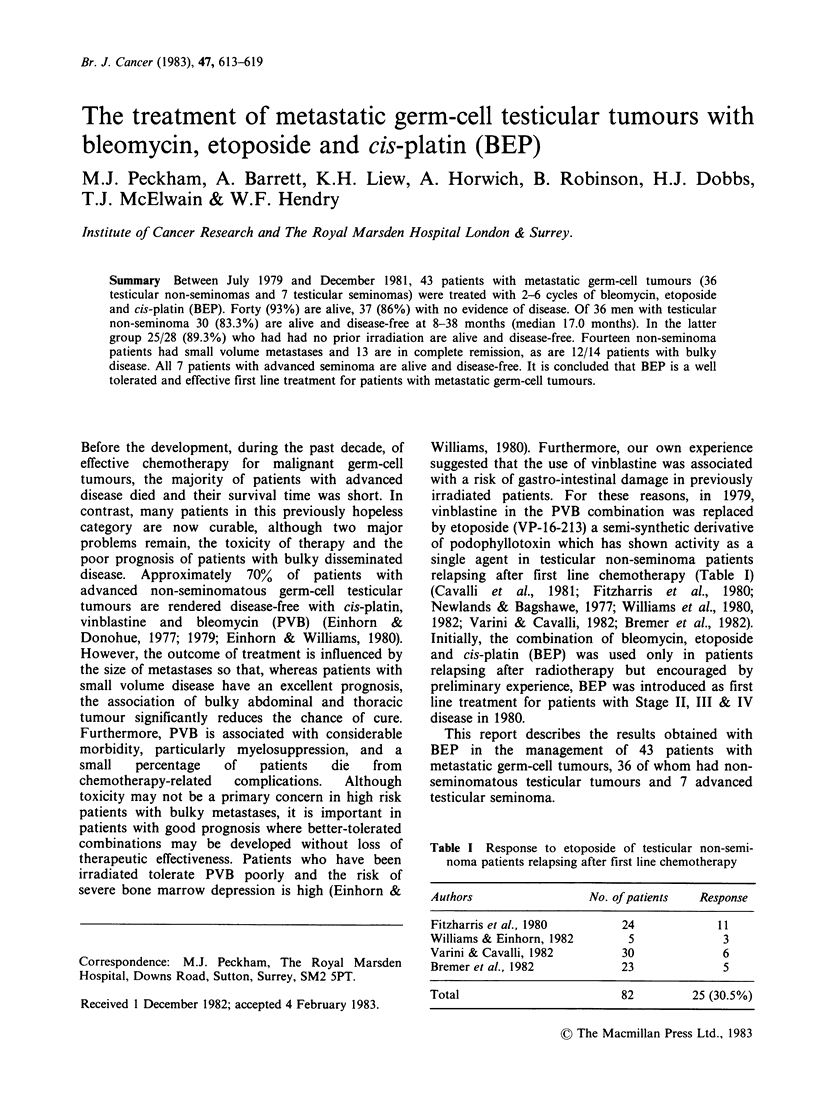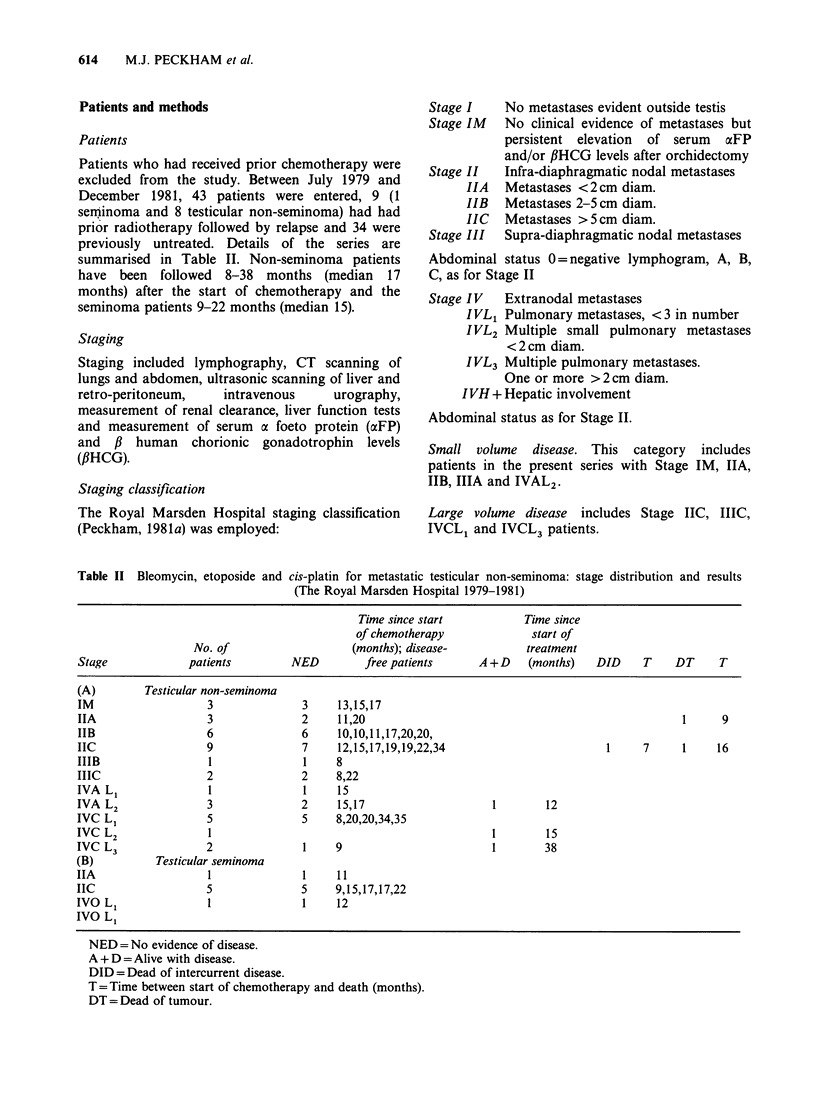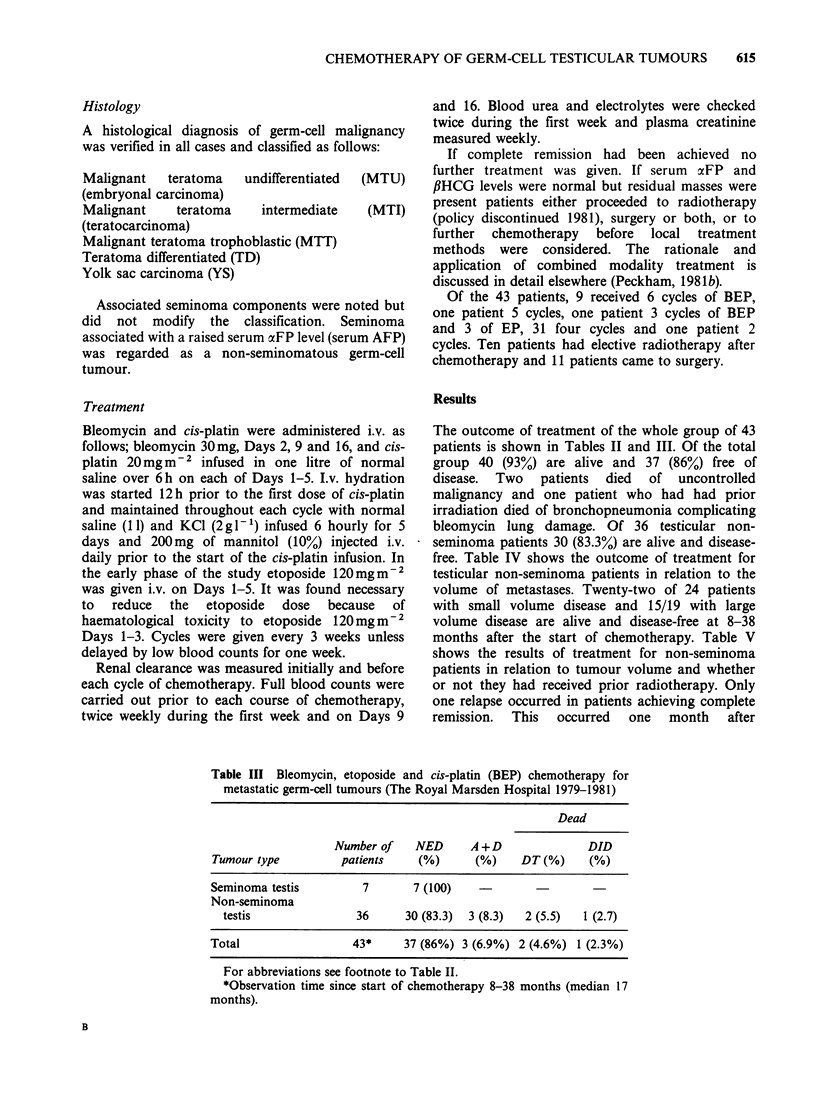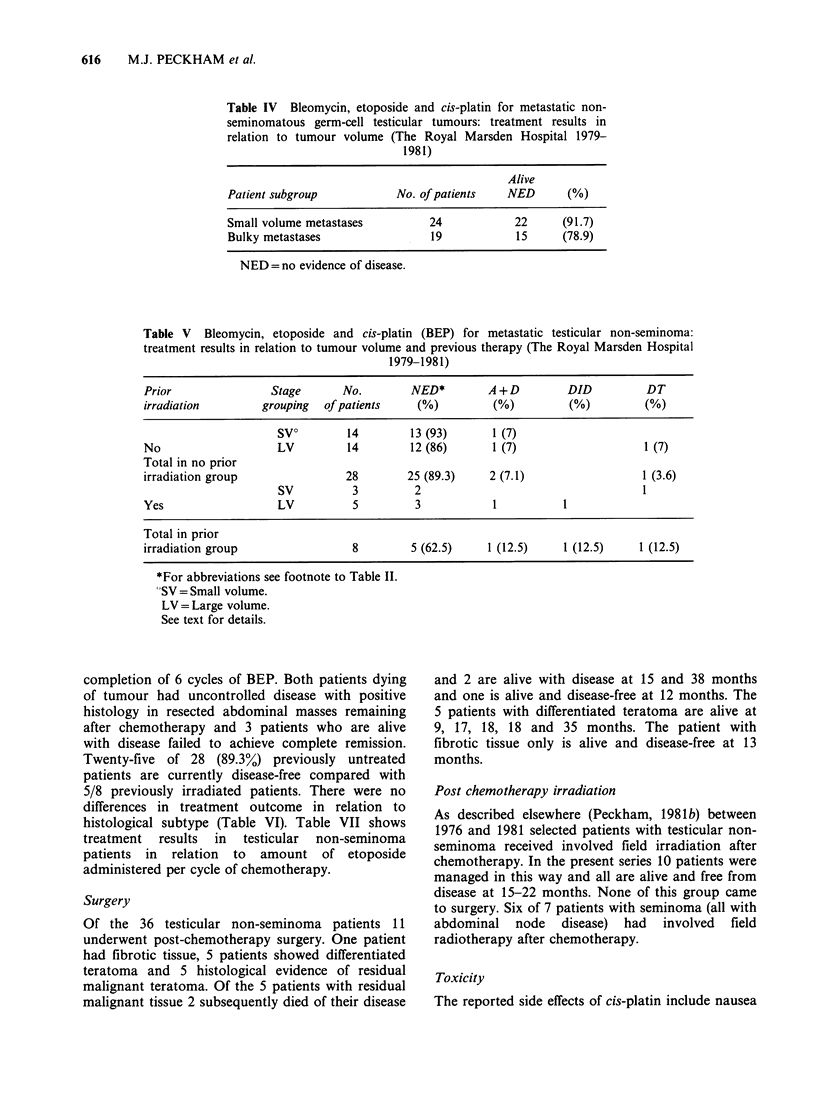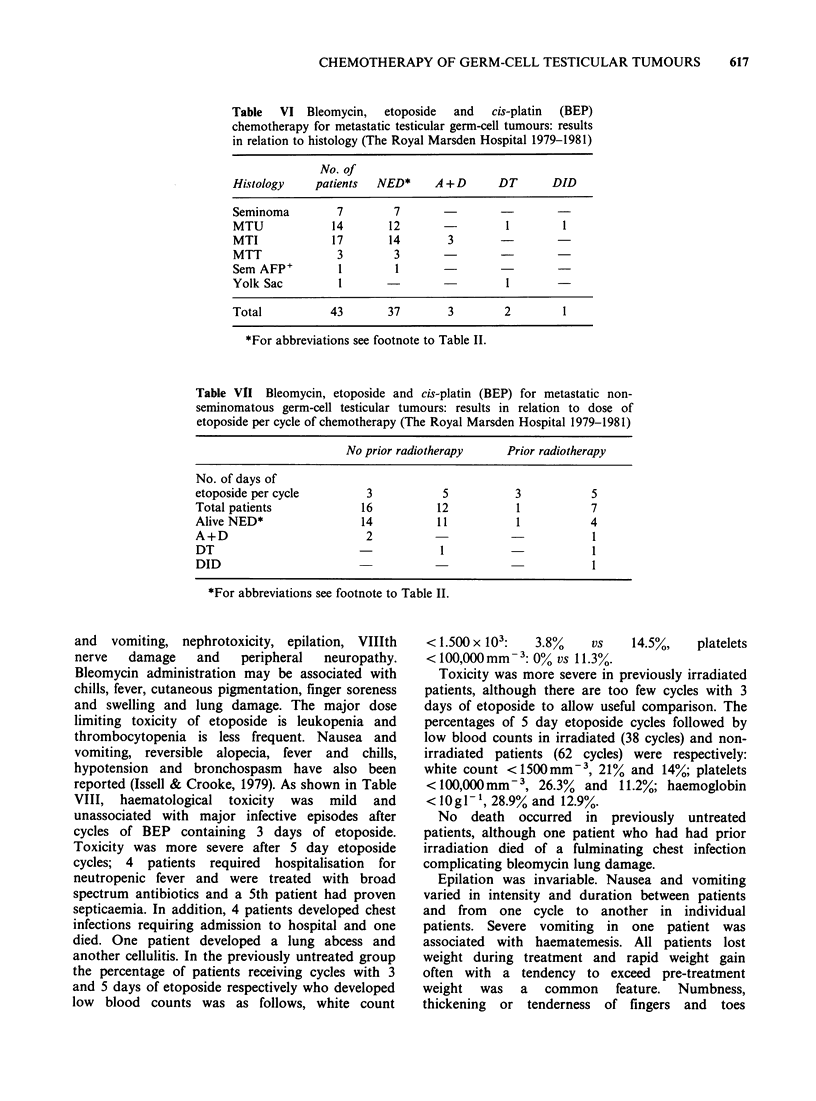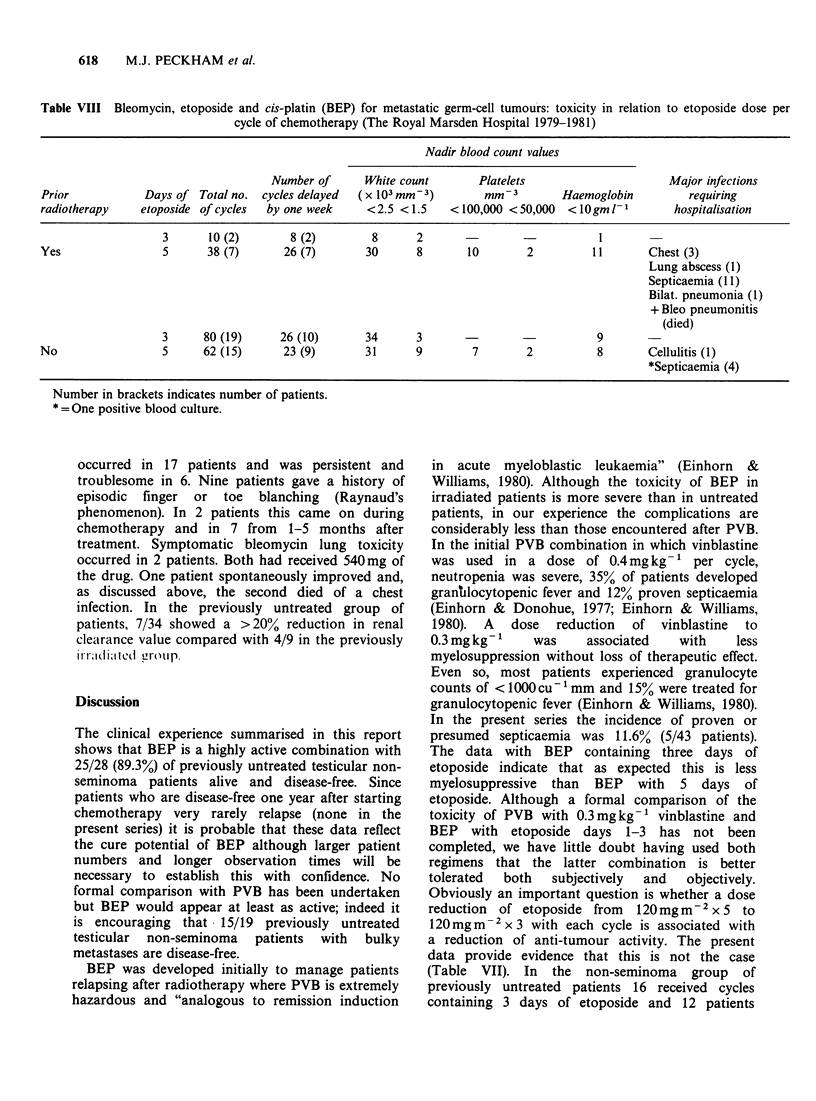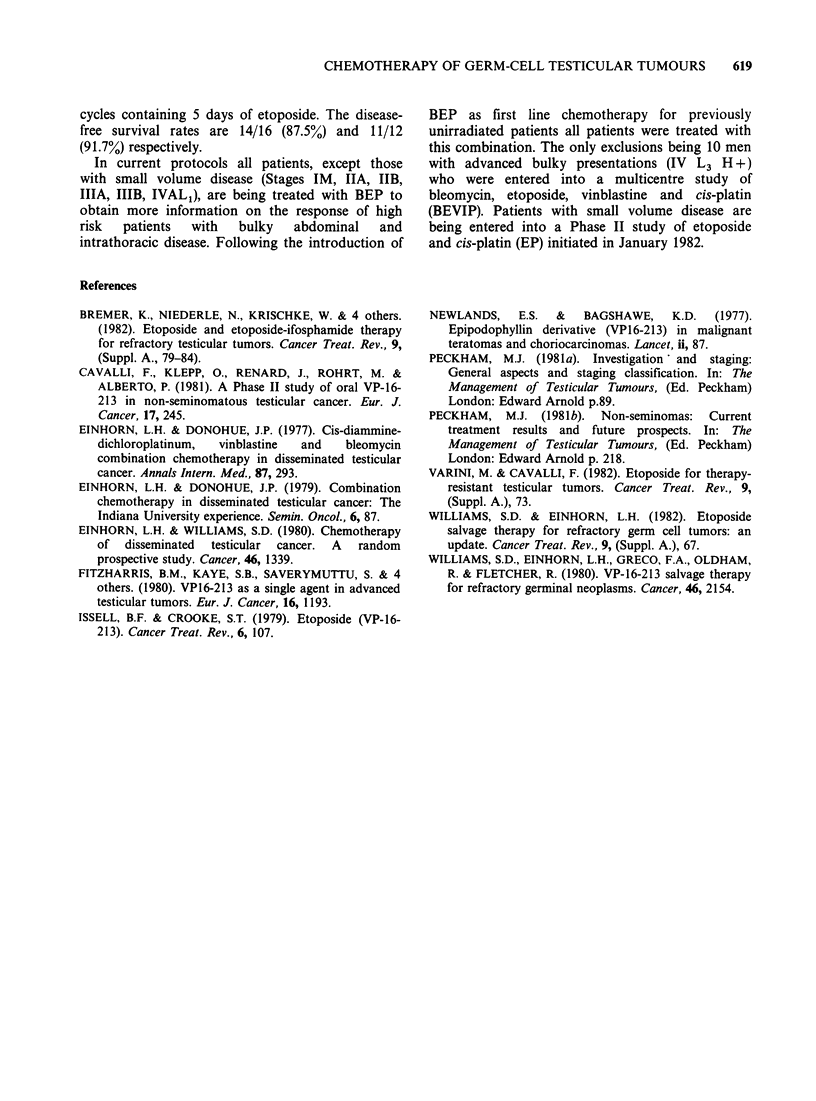# The treatment of metastatic germ-cell testicular tumours with bleomycin, etoposide and cis-platin (BEP).

**DOI:** 10.1038/bjc.1983.99

**Published:** 1983-05

**Authors:** M. J. Peckham, A. Barrett, K. H. Liew, A. Horwich, B. Robinson, H. J. Dobbs, T. J. McElwain, W. F. Hendry

## Abstract

Between July 1979 and December 1981, 43 patients with metastatic germ-cell tumours (36 testicular non-seminomas and 7 testicular seminomas) were treated with 2-6 cycles of bleomycin, etoposide and cis-platin (BEP). Forty (93%) are alive, 37 (86%) with no evidence of disease. Of 36 men with testicular non-seminoma 30 (83.3%) are alive and disease-free at 8-38 months (median 17.0 months). In the latter group 25/28 (89.3%) who had had no prior irradiation are alive and disease-free. Fourteen non-seminoma patients had small volume metastases and 13 are in complete remission, as are 12/14 patients with bulky disease. All 7 patients with advanced seminoma are alive and disease-free. It is concluded that BEP is a well tolerated and effective first line treatment for patients with metastatic germ-cell tumours.


*Br. J. Cancer* (1983), **47**, 613–619

### The treatment of metastatic germ-cell testicular tumours with bleomycin, etoposide and *cis*-platin (BEP)

M.J. Peckham, A. Barrett, K.H. Liew, A. Horwich, B. Robinson, H.J. Dobbs, T.J. McElwain & W.F. Hendry

*Institute of Cancer Research and The Royal Marsden Hospital London & Surrey.*

**Summary** Between July 1979 and December 1981, 43 patients with metastatic germ-cell tumours (36 testicular non-seminomas and 7 testicular seminomas) were treated with 2–6 cycles of bleomycin, etoposide and *cis*-platin (BEP). Forty (93%) are alive, 37 (86%) with no evidence of disease. Of 36 men with testicular non-seminoma 30 (83.3%) are alive and disease-free at 8–38 months (median 17.0 months). In the latter group 25/28 (89.3%) who had had no prior irradiation are alive and disease-free. Fourteen non-seminoma patients had small volume metastases and 13 are in complete remission, as are 12/14 patients with bulky disease. All 7 patients with advanced seminoma are alive and disease-free. It is concluded that BEP is a well tolerated and effective first line treatment for patients with metastatic germ-cell tumours.

Before the development, during the past decade, of effective chemotherapy for malignant germ-cell tumours, the majority of patients with advanced disease died and their survival time was short. In contrast, many patients in this previously hopeless category are now curable, although two major problems remain, the toxicity of therapy and the poor prognosis of patients with bulky disseminated disease. Approximately 70% of patients with advanced non-seminomatous germ-cell testicular tumours are rendered disease-free with *cis*-platin, vinblastine and bleomycin (PVB) (Einhorn & Donohue, 1977; 1979; Einhorn & Williams, 1980). However, the outcome of treatment is influenced by the size of metastases so that, whereas patients with small volume disease have an excellent prognosis, the association of bulky abdominal and thoracic tumour significantly reduces the chance of cure. Furthermore, PVB is associated with considerable morbidity, particularly myelosuppression, and a small percentage of patients die from chemotherapy-related complications. Although toxicity may not be a primary concern in high risk patients with bulky metastases, it is important in patients with good prognosis where better-tolerated combinations may be developed without loss of therapeutic effectiveness. Patients who have been irradiated tolerate PVB poorly and the risk of severe bone marrow depression is high (Einhorn & Williams, 1980). Furthermore, our own experience suggested that the use of vinblastine was associated with a risk of gastro-intestinal damage in previously irradiated patients. For these reasons, in 1979, vinblastine in the PVB combination was replaced by etoposide (VP-16-213) a semi-synthetic derivative of podophyllotoxin which has shown activity as a single agent in testicular non-seminoma patients relapsing after first line chemotherapy (Table I) (Cavalli *et al.*, 1981; Fitzharris *et al.*, 1980; Newlands & Bagshawe, 1977; Williams *et al.*, 1980, 1982; Varini & Cavalli, 1982; Bremer *et al.*, 1982). Initially, the combination of bleomycin, etoposide and *cis*-platin (BEP) was used only in patients relapsing after radiotherapy but encouraged by preliminary experience, BEP was introduced as first line treatment for patients with Stage II, III & IV disease in 1980.

This report describes the results obtained with BEP in the management of 43 patients with metastatic germ-cell tumours, 36 of whom had non-seminomatous testicular tumours and 7 advanced testicular seminoma.

**Table I** Response to etoposide of testicular non-seminoma patients relapsing after first line chemotherapy

| *Authors* | *No. of patients* | *Response* |
|---|---|---|
| Fitzharris *et al.*, 1980 | 24 | 11 |
| Williams & Einhorn, 1982 | 5 | 3 |
| Varini & Cavalli, 1982 | 30 | 6 |
| Bremer *et al.*, 1982 | 23 | 5 |
| Total | 82 | 25 (30.5%) |

Correspondence: M.J. Peckham, The Royal Marsden Hospital, Downs Road, Sutton, Surrey, SM2 5PT.

Received 1 December 1982; accepted 4 February 1983.

© The Macmillan Press Ltd., 1983

### Patients and methods

### *Patients*

Patients who had received prior chemotherapy were excluded from the study. Between July 1979 and December 1981, 43 patients were entered, 9 (1 seminoma and 8 testicular non-seminoma) had had prior radiotherapy followed by relapse and 34 were previously untreated. Details of the series are summarised in Table II. Non-seminoma patients have been followed 8–38 months (median 17 months) after the start of chemotherapy and the seminoma patients 9–22 months (median 15).

### *Staging*

Staging included lymphography, CT scanning of lungs and abdomen, ultrasonic scanning of liver and retro-peritoneum, intravenous urography, measurement of renal clearance, liver function tests and measurement of serum α foeto protein (αFP) and β human chorionic gonadotrophin levels (βHCG).

### *Staging classification*

The Royal Marsden Hospital staging classification (Peckham, 1981*a*) was employed:

*Stage I* No metastases evident outside testis
*Stage IM* No clinical evidence of metastases but persistent elevation of serum αFP and/or βHCG levels after orchidectomy
*Stage II* Infra-diaphragmatic nodal metastases
*IIA* Metastases $<2$ cm diam.
*IIB* Metastases 2–5 cm diam.
*IIC* Metastases $>5$ cm diam.
*Stage III* Supra-diaphragmatic nodal metastases

Abdominal status 0 = negative lymphogram, A, B, C, as for Stage II

*Stage IV* Extranodal metastases
$IVL_1$ Pulmonary metastases, $<3$ in number
$IVL_2$ Multiple small pulmonary metastases $<2$ cm diam.
$IVL_3$ Multiple pulmonary metastases. One or more $>2$ cm diam.
$IVH+$ Hepatic involvement

Abdominal status as for Stage II.

*Small volume disease.* This category includes patients in the present series with Stage IM, IIA, IIB, IIIA and $IVAL_2$.

*Large volume disease* includes Stage IIC, IIIC, $IVCL_1$ and $IVCL_3$ patients.

**Table II** Bleomycin, etoposide and *cis*-platin for metastatic testicular non-seminoma: stage distribution and results (The Royal Marsden Hospital 1979–1981)

| *Stage* | *No. of patients* | *NED* | *Time since start of chemotherapy (months); disease-free patients* | *A+D* | *Time since start of treatment (months)* | *DID* | *T* | *DT* | *T* |
|---|---|---|---|---|---|---|---|---|---|
| (A) | *Testicular non-seminoma* | | | | | | | | |
| IM | 3 | 3 | 13,15,17 | | | | | | |
| IIA | 3 | 2 | 11,20 | | | | | 1 | 9 |
| IIB | 6 | 6 | 10,10,11,17,20,20, | | | | | | |
| IIC | 9 | 7 | 12,15,17,19,19,22,34 | | | 1 | 7 | 1 | 16 |
| IIIB | 1 | 1 | 8 | | | | | | |
| IIIC | 2 | 2 | 8,22 | | | | | | |
| IVA $L_1$ | 1 | 1 | 15 | | | | | | |
| IVA $L_2$ | 3 | 2 | 15,17 | 1 | 12 | | | | |
| IVC $L_1$ | 5 | 5 | 8,20,20,34,35 | | | | | | |
| IVC $L_2$ | 1 | | | 1 | 15 | | | | |
| IVC $L_3$ | 2 | 1 | 9 | 1 | 38 | | | | |
| (B) | *Testicular seminoma* | | | | | | | | |
| IIA | 1 | 1 | 11 | | | | | | |
| IIC | 5 | 5 | 9,15,17,17,22 | | | | | | |
| IVO $L_1$ | 1 | 1 | 12 | | | | | | |
| IVO $L_1$ | | | | | | | | | |

NED = No evidence of disease.
A + D = Alive with disease.
DID = Dead of intercurrent disease.
T = Time between start of chemotherapy and death (months).
DT = Dead of tumour.

*Histology*

A histological diagnosis of germ-cell malignancy was verified in all cases and classified as follows:

Malignant teratoma undifferentiated (MTU) (embryonal carcinoma)
Malignant teratoma intermediate (MTI) (teratocarcinoma)
Malignant teratoma trophoblastic (MTT)
Teratoma differentiated (TD)
Yolk sac carcinoma (YS)

Associated seminoma components were noted but did not modify the classification. Seminoma associated with a raised serum αFP level (serum AFP) was regarded as a non-seminomatous germ-cell tumour.

*Treatment*

Bleomycin and *cis*-platin were administered i.v. as follows; bleomycin 30 mg, Days 2, 9 and 16, and *cis*-platin 20 mg $m^{-2}$ infused in one litre of normal saline over 6 h on each of Days 1–5. I.v. hydration was started 12 h prior to the first dose of *cis*-platin and maintained throughout each cycle with normal saline (1 l) and KCl (2 g $l^{-1}$) infused 6 hourly for 5 days and 200 mg of mannitol (10%) injected i.v. daily prior to the start of the *cis*-platin infusion. In the early phase of the study etoposide 120 mg $m^{-2}$ was given i.v. on Days 1–5. It was found necessary to reduce the etoposide dose because of haematological toxicity to etoposide 120 mg $m^{-2}$ Days 1–3. Cycles were given every 3 weeks unless delayed by low blood counts for one week.

Renal clearance was measured initially and before each cycle of chemotherapy. Full blood counts were carried out prior to each course of chemotherapy, twice weekly during the first week and on Days 9 and 16. Blood urea and electrolytes were checked twice during the first week and plasma creatinine measured weekly.

If complete remission had been achieved no further treatment was given. If serum αFP and βHCG levels were normal but residual masses were present patients either proceeded to radiotherapy (policy discontinued 1981), surgery or both, or to further chemotherapy before local treatment methods were considered. The rationale and application of combined modality treatment is discussed in detail elsewhere (Peckham, 1981*b*).

Of the 43 patients, 9 received 6 cycles of BEP, one patient 5 cycles, one patient 3 cycles of BEP and 3 of EP, 31 four cycles and one patient 2 cycles. Ten patients had elective radiotherapy after chemotherapy and 11 patients came to surgery.

### Results

The outcome of treatment of the whole group of 43 patients is shown in Tables II and III. Of the total group 40 (93%) are alive and 37 (86%) free of disease. Two patients died of uncontrolled malignancy and one patient who had had prior irradiation died of bronchopneumonia complicating bleomycin lung damage. Of 36 testicular non-seminoma patients 30 (83.3%) are alive and disease-free. Table IV shows the outcome of treatment for testicular non-seminoma patients in relation to the volume of metastases. Twenty-two of 24 patients with small volume disease and 15/19 with large volume disease are alive and disease-free at 8–38 months after the start of chemotherapy. Table V shows the results of treatment for non-seminoma patients in relation to tumour volume and whether or not they had received prior radiotherapy. Only one relapse occurred in patients achieving complete remission. This occurred one month after

**Table III** Bleomycin, etoposide and *cis*-platin (BEP) chemotherapy for metastatic germ-cell tumours (The Royal Marsden Hospital 1979–1981)

| | | | | Dead | |
|---|---|---|---|---|---|
| *Tumour type* | *Number of patients* | *NED* (%) | *A+D* (%) | *DT* (%) | *DID* (%) |
| Seminoma testis | 7 | 7 (100) | — | — | — |
| Non-seminoma testis | 36 | 30 (83.3) | 3 (8.3) | 2 (5.5) | 1 (2.7) |
| Total | 43* | 37 (86%) | 3 (6.9%) | 2 (4.6%) | 1 (2.3%) |

For abbreviations see footnote to Table II.

*Observation time since start of chemotherapy 8–38 months (median 17 months).

**Table IV** Bleomycin, etoposide and *cis*-platin for metastatic non-seminomatous germ-cell testicular tumours: treatment results in relation to tumour volume (The Royal Marsden Hospital 1979–1981)

| *Patient subgroup* | *No. of patients* | *Alive NED* | (%) |
|---|---|---|---|
| Small volume metastases | 24 | 22 | (91.7) |
| Bulky metastases | 19 | 15 | (78.9) |

NED = no evidence of disease.

**Table V** Bleomycin, etoposide and *cis*-platin (BEP) for metastatic testicular non-seminoma: treatment results in relation to tumour volume and previous therapy (The Royal Marsden Hospital 1979–1981)

| *Prior irradiation* | *Stage grouping* | *No. of patients* | *NED** (%) | *A+D* (%) | *DID* (%) | *DT* (%) |
|---|---|---|---|---|---|---|
| | SV° | 14 | 13 (93) | 1 (7) | | |
| No | LV | 14 | 12 (86) | 1 (7) | | 1 (7) |
| Total in no prior irradiation group | | 28 | 25 (89.3) | 2 (7.1) | | 1 (3.6) |
| | SV | 3 | 2 | | | 1 |
| Yes | LV | 5 | 3 | 1 | 1 | |
| Total in prior irradiation group | | 8 | 5 (62.5) | 1 (12.5) | 1 (12.5) | 1 (12.5) |

*For abbreviations see footnote to Table II.
°SV = Small volume.
LV = Large volume.
See text for details.

completion of 6 cycles of BEP. Both patients dying of tumour had uncontrolled disease with positive histology in resected abdominal masses remaining after chemotherapy and 3 patients who are alive with disease failed to achieve complete remission. Twenty-five of 28 (89.3%) previously untreated patients are currently disease-free compared with 5/8 previously irradiated patients. There were no differences in treatment outcome in relation to histological subtype (Table VI). Table VII shows treatment results in testicular non-seminoma patients in relation to amount of etoposide administered per cycle of chemotherapy.

*Surgery*

Of the 36 testicular non-seminoma patients 11 underwent post-chemotherapy surgery. One patient had fibrotic tissue, 5 patients showed differentiated teratoma and 5 histological evidence of residual malignant teratoma. Of the 5 patients with residual malignant tissue 2 subsequently died of their disease and 2 are alive with disease at 15 and 38 months and one is alive and disease-free at 12 months. The 5 patients with differentiated teratoma are alive at 9, 17, 18, 18 and 35 months. The patient with fibrotic tissue only is alive and disease-free at 13 months.

*Post chemotherapy irradiation*

As described elsewhere (Peckham, 1981*b*) between 1976 and 1981 selected patients with testicular non-seminoma received involved field irradiation after chemotherapy. In the present series 10 patients were managed in this way and all are alive and free from disease at 15–22 months. None of this group came to surgery. Six of 7 patients with seminoma (all with abdominal node disease) had involved field radiotherapy after chemotherapy.

*Toxicity*

The reported side effects of *cis*-platin include nausea

**Table VI** Bleomycin, etoposide and *cis*-platin (BEP) chemotherapy for metastatic testicular germ-cell tumours: results in relation to histology (The Royal Marsden Hospital 1979–1981)

| *Histology* | *No. of patients* | *NED** | *A+D* | *DT* | *DID* |
|---|---|---|---|---|---|
| Seminoma | 7 | 7 | — | — | — |
| MTU | 14 | 12 | — | 1 | 1 |
| MTI | 17 | 14 | 3 | — | — |
| MTT | 3 | 3 | — | — | — |
| Sem AFP$^+$ | 1 | 1 | — | — | — |
| Yolk Sac | 1 | — | — | 1 | — |
| Total | 43 | 37 | 3 | 2 | 1 |

*For abbreviations see footnote to Table II.

**Table VII** Bleomycin, etoposide and *cis*-platin (BEP) for metastatic non-seminomatous germ-cell testicular tumours: results in relation to dose of etoposide per cycle of chemotherapy (The Royal Marsden Hospital 1979–1981)

| | *No prior radiotherapy* | | *Prior radiotherapy* | |
|---|---|---|---|---|
| No. of days of etoposide per cycle | 3 | 5 | 3 | 5 |
| Total patients | 16 | 12 | 1 | 7 |
| Alive NED* | 14 | 11 | 1 | 4 |
| A+D | 2 | — | — | 1 |
| DT | — | 1 | — | 1 |
| DID | — | — | — | 1 |

*For abbreviations see footnote to Table II.

and vomiting, nephrotoxicity, epilation, VIIIth nerve damage and peripheral neuropathy. Bleomycin administration may be associated with chills, fever, cutaneous pigmentation, finger soreness and swelling and lung damage. The major dose limiting toxicity of etoposide is leukopenia and thrombocytopenia is less frequent. Nausea and vomiting, reversible alopecia, fever and chills, hypotension and bronchospasm have also been reported (Issell & Crooke, 1979). As shown in Table VIII, haematological toxicity was mild and unassociated with major infective episodes after cycles of BEP containing 3 days of etoposide. Toxicity was more severe after 5 day etoposide cycles; 4 patients required hospitalisation for neutropenic fever and were treated with broad spectrum antibiotics and a 5th patient had proven septicaemia. In addition, 4 patients developed chest infections requiring admission to hospital and one died. One patient developed a lung abcess and another cellulitis. In the previously untreated group the percentage of patients receiving cycles with 3 and 5 days of etoposide respectively who developed low blood counts was as follows, white count $<1.500\times10^3$: 3.8% *vs* 14.5%, platelets $<100{,}000\,\mathrm{mm}^{-3}$: 0% *vs* 11.3%.

Toxicity was more severe in previously irradiated patients, although there are too few cycles with 3 days of etoposide to allow useful comparison. The percentages of 5 day etoposide cycles followed by low blood counts in irradiated (38 cycles) and non-irradiated patients (62 cycles) were respectively: white count $<1500\,\mathrm{mm}^{-3}$, 21% and 14%; platelets $<100{,}000\,\mathrm{mm}^{-3}$, 26.3% and 11.2%; haemoglobin $<10\,\mathrm{g\,l}^{-1}$, 28.9% and 12.9%.

No death occurred in previously untreated patients, although one patient who had had prior irradiation died of a fulminating chest infection complicating bleomycin lung damage.

Epilation was invariable. Nausea and vomiting varied in intensity and duration between patients and from one cycle to another in individual patients. Severe vomiting in one patient was associated with haematemesis. All patients lost weight during treatment and rapid weight gain often with a tendency to exceed pre-treatment weight was a common feature. Numbness, thickening or tenderness of fingers and toes

**Table VIII** Bleomycin, etoposide and *cis*-platin (BEP) for metastatic germ-cell tumours: toxicity in relation to etoposide dose per cycle of chemotherapy (The Royal Marsden Hospital 1979–1981)

| | | | | Nadir blood count values | | | | | |
|---|---|---|---|---|---|---|---|---|---|
| *Prior radiotherapy* | *Days of etoposide* | *Total no. of cycles* | *Number of cycles delayed by one week* | *White count* ($\times 10^3\ mm^{-3}$) $<2.5$ | $<1.5$ | *Platelets* $mm^{-3}$ $<100,000$ | $<50,000$ | *Haemoglobin* $<10\ g\,ml^{-1}$ | *Major infections requiring hospitalisation* |
| | 3 | 10 (2) | 8 (2) | 8 | 2 | — | — | 1 | — |
| Yes | 5 | 38 (7) | 26 (7) | 30 | 8 | 10 | 2 | 11 | Chest (3)<br>Lung abscess (1)<br>Septicaemia (11)<br>Bilat. pneumonia (1)<br>+Bleo pneumonitis (died) |
| | 3 | 80 (19) | 26 (10) | 34 | 3 | — | — | 9 | — |
| No | 5 | 62 (15) | 23 (9) | 31 | 9 | 7 | 2 | 8 | Cellulitis (1)<br>*Septicaemia (4) |

Number in brackets indicates number of patients.
* = One positive blood culture.

occurred in 17 patients and was persistent and troublesome in 6. Nine patients gave a history of episodic finger or toe blanching (Raynaud's phenomenon). In 2 patients this came on during chemotherapy and in 7 from 1–5 months after treatment. Symptomatic bleomycin lung toxicity occurred in 2 patients. Both had received 540 mg of the drug. One patient spontaneously improved and, as discussed above, the second died of a chest infection. In the previously untreated group of patients, 7/34 showed a >20% reduction in renal clearance value compared with 4/9 in the previously irradiated group.

### Discussion

The clinical experience summarised in this report shows that BEP is a highly active combination with 25/28 (89.3%) of previously untreated testicular non-seminoma patients alive and disease-free. Since patients who are disease-free one year after starting chemotherapy very rarely relapse (none in the present series) it is probable that these data reflect the cure potential of BEP although larger patient numbers and longer observation times will be necessary to establish this with confidence. No formal comparison with PVB has been undertaken but BEP would appear at least as active; indeed it is encouraging that 15/19 previously untreated testicular non-seminoma patients with bulky metastases are disease-free.

BEP was developed initially to manage patients relapsing after radiotherapy where PVB is extremely hazardous and "analogous to remission induction in acute myeloblastic leukaemia" (Einhorn & Williams, 1980). Although the toxicity of BEP in irradiated patients is more severe than in untreated patients, in our experience the complications are considerably less than those encountered after PVB. In the initial PVB combination in which vinblastine was used in a dose of $0.4\,mg\,kg^{-1}$ per cycle, neutropenia was severe, 35% of patients developed granulocytopenic fever and 12% proven septicaemia (Einhorn & Donohue, 1977; Einhorn & Williams, 1980). A dose reduction of vinblastine to $0.3\,mg\,kg^{-1}$ was associated with less myelosuppression without loss of therapeutic effect. Even so, most patients experienced granulocyte counts of $<1000\,cu^{-1}\,mm$ and 15% were treated for granulocytopenic fever (Einhorn & Williams, 1980). In the present series the incidence of proven or presumed septicaemia was 11.6% (5/43 patients). The data with BEP containing three days of etoposide indicate that as expected this is less myelosuppressive than BEP with 5 days of etoposide. Although a formal comparison of the toxicity of PVB with $0.3\,mg\,kg^{-1}$ vinblastine and BEP with etoposide days 1–3 has not been completed, we have little doubt having used both regimens that the latter combination is better tolerated both subjectively and objectively. Obviously an important question is whether a dose reduction of etoposide from $120\,mg\,m^{-2} \times 5$ to $120\,mg\,m^{-2} \times 3$ with each cycle is associated with a reduction of anti-tumour activity. The present data provide evidence that this is not the case (Table VII). In the non-seminoma group of previously untreated patients 16 received cycles containing 3 days of etoposide and 12 patients cycles containing 5 days of etoposide. The disease-free survival rates are 14/16 (87.5%) and 11/12 (91.7%) respectively.

In current protocols all patients, except those with small volume disease (Stages IM, IIA, IIB, IIIA, IIIB, $IVAL_1$), are being treated with BEP to obtain more information on the response of high risk patients with bulky abdominal and intrathoracic disease. Following the introduction of BEP as first line chemotherapy for previously unirradiated patients all patients were treated with this combination. The only exclusions being 10 men with advanced bulky presentations (IV $L_3$ H+) who were entered into a multicentre study of bleomycin, etoposide, vinblastine and *cis*-platin (BEVIP). Patients with small volume disease are being entered into a Phase II study of etoposide and *cis*-platin (EP) initiated in January 1982.

### References

BREMER, K., NIEDERLE, N., KRISCHKE, W. & 4 others. (1982). Etoposide and etoposide-ifosphamide therapy for refractory testicular tumors. *Cancer Treat. Rev.*, **9**, (Suppl. A., 79–84).

CAVALLI, F., KLEPP, O., RENARD, J., ROHRT, M. & ALBERTO, P. (1981). A Phase II study of oral VP-16-213 in non-seminomatous testicular cancer. *Eur. J. Cancer*, **17**, 245.

EINHORN, L.H. & DONOHUE, J.P. (1977). Cis-diammine-dichloroplatinum, vinblastine and bleomycin combination chemotherapy in disseminated testicular cancer. *Annals Intern. Med.*, **87**, 293.

EINHORN, L.H. & DONOHUE, J.P. (1979). Combination chemotherapy in disseminated testicular cancer: The Indiana University experience. *Semin. Oncol.*, **6**, 87.

EINHORN, L.H. & WILLIAMS, S.D. (1980). Chemotherapy of disseminated testicular cancer. A random prospective study. *Cancer*, **46**, 1339.

FITZHARRIS, B.M., KAYE, S.B., SAVERYMUTTU, S. & 4 others. (1980). VP16-213 as a single agent in advanced testicular tumors. *Eur. J. Cancer*, **16**, 1193.

ISSELL, B.F. & CROOKE, S.T. (1979). Etoposide (VP-16-213). *Cancer Treat. Rev.*, **6**, 107.

NEWLANDS, E.S. & BAGSHAWE, K.D. (1977). Epipodophyllin derivative (VP16-213) in malignant teratomas and choriocarcinomas. *Lancet*, **ii**, 87.

PECKHAM, M.J. (1981*a*). Investigation ' and staging: General aspects and staging classification. In: *The Management of Testicular Tumours*, (Ed. Peckham) London: Edward Arnold p.89.

PECKHAM, M.J. (1981*b*). Non-seminomas: Current treatment results and future prospects. In: *The Management of Testicular Tumours*, (Ed. Peckham) London: Edward Arnold p. 218.

VARINI, M. & CAVALLI, F. (1982). Etoposide for therapy-resistant testicular tumors. *Cancer Treat. Rev.*, **9**, (Suppl. A.), 73.

WILLIAMS, S.D. & EINHORN, L.H. (1982). Etoposide salvage therapy for refractory germ cell tumors: an update. *Cancer Treat. Rev.*, **9**, (Suppl. A.), 67.

WILLIAMS, S.D., EINHORN, L.H., GRECO, F.A., OLDHAM, R. & FLETCHER, R. (1980). VP-16-213 salvage therapy for refractory germinal neoplasms. *Cancer*, **46**, 2154.